# Access to Routine Immunization: A Comparative Analysis of Supply-Side Disparities between Northern and Southern Nigeria

**DOI:** 10.1371/journal.pone.0144876

**Published:** 2015-12-21

**Authors:** Ejemai Eboreime, Seye Abimbola, Fiammetta Bozzani

**Affiliations:** 1 Department of Planning, Research and Statistics, National Primary Health Care Development Agency, Abuja, Nigeria; 2 School of Public Health, Sydney Medical School, University of Sydney, Sydney, Australia; 3 Department of Global Health and Development, London School of Hygiene and Tropical Medicine, London, United Kingdom; Centre Hospitalier Universitaire Vaudois, FRANCE

## Abstract

**Background:**

The available data on routine immunization in Nigeria show a disparity in coverage between Northern and Southern Nigeria, with the former performing worse. The effect of socio-cultural differences on health-seeking behaviour has been identified in the literature as the main cause of the disparity. Our study analyses the role of supply-side determinants, particularly access to services, in causing these disparities.

**Methods:**

Using routine government data, we compared supply-side determinants of access in two Northern states with two Southern states. The states were identified using criteria-based purposive selection such that the comparisons were made between a low-coverage state in the South and a low-coverage state in the North as well as between a high-coverage state in the South and a high-coverage state in the North.

**Results:**

Human resources and commodities at routine immunization service delivery points were generally insufficient for service delivery in both geographical regions. While disparities were evident between individual states irrespective of regional location, compared to the South, residents in Northern Nigeria were more likely to have vaccination service delivery points located within a 5km radius of their settlements.

**Conclusion:**

Our findings suggest that regional supply-side disparities are not apparent, reinforcing the earlier reported socio-cultural explanations for disparities in routine immunization service uptake between Northern and Southern Nigeria. Nonetheless, improving routine immunisation coverage services require that there are available human resources and that health facilities are equitably distributed.

## Introduction

Ever since the demonstration of the value of immunization by Edward Jenner in 1792, vaccination has increasingly become a key strategy in the prevention and control of infectious diseases globally. Nigeria, with a population of over 170 million, has one of the highest under five mortality rates in the world (143 deaths per 1000 live births), with about 25% of these deaths preventable through routine immunization [1]. Available data for routine immunization show a disparity in coverage between Northern and Southern Nigeria. National surveys reveal that, compared to the South, Northern Nigeria performs poorly on health indices, including immunization coverage[2]. Previous studies show that children in parts of Southern Nigeria have more than twice the chances of receiving full immunization compared to children in parts of Northern Nigeria [3].

Nigeria is comprised of 37 states (inclusive of the Federal Capital Territory) which in turn comprise a total of 774 Local Government Areas (LGAs). The constitution of Nigeria creates a considerably decentralised system of government (politically and fiscally) in which health is the concurrent responsibility of the federal, state and local tiers of government [4, 5]. The federal government develops policies and guidelines, provides technical support and is responsible for the delivery of tertiary health care, while secondary and primary health care (PHC) are under the purview of the states and local governments, respectively. In relation to immunisation, the federal government purchases vaccines, develops immunisation guidelines and provides technical support to sub-national governments through the National PHC Development Agency. However, states and local governments provide infrastructure and logistics to deliver routine immunization services. The policy of the government of Nigeria is to *“provide immunization services and potent vaccines free to all population at risk”* [6]. And to ensure equitable access, the federal government recommends having routine immunisation services available within 5km of any community [6, 7]. However, a 2013 survey found that immunisation coverage in Northern Nigeria zones ranged from 14% to 44%, while in Southern Nigeria zones, the range was 70% to 81% [2].

Northern Nigeria, which is predominantly Islamic, bears cultural semblance to the Arab states of North Africa and the Middle East [8, 9]. Although diverse in ethnicity, the Hausa and Fulani (a largely nomadic group) cultures predominate. Historically, Northern Nigeria is divided into Emirates, which are ruled by Fulani Emirs to whom minority groups paid tributes and from whom religious and social norms derive. Islamic education is still widespread in northern Nigeria [10]. Southern Nigeria on the other hand, with its numerous ethnic groups (the largest being the Yoruba, Igbo and Ijaw), has Christianity as its dominant religion. Southern Nigerians tend to embrace Western culture and education as a consequence of direct British colonial rule [8, 9]. Studies document the impact of these socio-political and ethno-religious diversities on health disparities, through their effect on health-seeking behaviour and other demand-side factors [3, 11]. Although little is known about regional disparities in supply-side determinants, researchers and policymakers have established and focussed on the association between demand-side factors and immunisation coverage. This evidence and focus has led to interventions such as education (including maternal education), advocacy and community mobilization as a means of addressing inequities in utilization of routine immunisation services [1, 3, 11]. In this study, we sought to examine the contribution of supply-side issues to inequities in routine immunization services between Northern and Southern Nigeria. **Our study hypothesis is that regional variations in vaccination uptake are not exclusively explained by the socioeconomic and cultural differences between the regions, but can also be attributed to supply-side determinants. We are thus looking at differences between high- and low-coverage states in the same region, where demand-side determinants are similar, to assess whether the same inequalities in access are uniformly present in the North and in the South.**

## Methods

### Study Setting

In this cross-sectional study, we compared equity in access in two Northern and two Southern Nigerian states. These four states were identified using purposive selection based on relative immunisation coverage and socio-cultural characteristics. The aim was to achieve a sample that was representative of the two regions in terms of culture and religion, with one Northern and one Southern state with a relatively high routine immunisation coverage, and one Northern and one Southern state with a relatively low routine immunisation coverage. Given that data for this study was obtained as part of the national Primary Health Care (PHC) reviews, states and local government areas which had not conducted PHC reviews for the third quarter of 2013 (i.e. July-September) were excluded. Also excluded were states and local governments which conducted the reviews but did not follow the guidelines, resulting in poor quality. Based on these criteria and using routine immunisation coverage data from the 2010 Nigeria Immunization Coverage Survey (NICS), the states of Abia, Ondo, Jigawa and Kano were selected (Table 1). Full immunisation in the 2010 NICS was defined as having “received doses of the standard six antigens—BCG, diphtheria–tetanus– pertussis (DTP) (3 doses), polio (3 doses), and measles vaccines” [12]. The wide variation in data available from various surveys informed our preference for the NICS given the immunization-specific nature of the survey. The NICS provides verifiable data on full immunisation obtained from vaccination cards, thus eliminating recall bias when compared to the more recent 2013 Nigeria Demographic and Health Survey in which information was obtained from either mother’s history or vaccination cards [2].

**Table 1:** Characteristics of States selected for study.

State	Region	Major Ethnic Groups	Dominant Religion	GDP per Capita	Immunisation Coverage
Abia	Southern Nigeria	Igbo	Christianity	Abia $3,003	8%
Ondo	Southern Nigeria	Yoruba, Ijaw	Christianity	Ondo $2,392	54%
Jigawa	Northern Nigeria	Hausa, Fulani, Kanuri	Islam	Jigawa $673	63%
Kano	Northern Nigeria	Hausa, Fulani	Islam	Kano $1,288	3.4%

Sources: [13, 12, 14].

### Data collection

The data used in this study were collected as part of national PHC reviews. These reviews were instituted in 2011 to monitor the implementation of the 2010–2015 National Strategic Health Development Plan [15]. The PHC reviews are conducted quarterly at the local government level, biannually at the state level and annually at the federal level. Data for the PHC reviews are obtained from the Health Management Information System (HMIS) summary forms and local government health plans. A modified Tanahashi model for health systems bottleneck analysis is used during the PHC reviews to assess bottlenecks at the LGA level, focusing on three supply-side determinants of effective coverage—availability of essential health commodities, availability of human resources for health, accessibility of distribution points for the interventions i.e. geographical access [7]. We used these supply side data from the PHC reviews, population distribution data, and routine data at the NPHCDA and the Federal Ministry of Health of Nigeria (Table 2).

**Table 2:** Data Sources.

S/N	Information	Resource Type	Source
1	Distribution of healthcare facilities in Nigeria	GIS data	NPHCDA Library
2	Distribution of human resources across health facilities	Published data (for some states)	NPHCDA
3	Immunization coverage	Published survey and Administrative reports	NPHCDA
4	Supply of health commodities	Unpublished	NPHCDA
5	Local Government Immunization micro-plans	Unpublished	NPHCDA
6	Immunization related reports, other research works, policy documents	Grey Literature	Internet, NPHCDA, FMOH, NPC

Abbreviations: GIS-Geographic Information System; NPHCDA- National Primary Healthcare Development Agency; FMOH- Federal Ministry of Health; NPC-National Population commission.

### Data analysis

Data from all health facilities and local governments involved in the PHC reviews in the selected states were analysed in the study. We compared the proportion of health facilities with trained health workers for routine immunisation services between Northern and Southern Nigeria. The proportion of facilities reporting stock-outs of vaccines was also compared across the regions. The proportion of target population living within a 5km radius of routine immunisation service delivery points in each state was determined by analysing data aggregated at the local government level within the state. Comparisons were made between the low-coverage state in the South and the low-coverage state in the North as well as between the high-coverage state in the South and the high-coverage state in the North. Two-by-two contingency tables were developed to analyse the relationship between geographical location and the indicator variables, i.e. availability of commodities, distance from service delivery points and availability of trained routine immunisation service providers. Fisher’s Exact Test calculated using STATA was used to assess the evidence of an association between the indicator variables (outcome) and residing in Northern or Southern Nigeria (exposure variable). Prevalence ratios were calculated for those indicators showing significant evidence of an association with the exposure variable using STATA. Prevalence ratios were used instead of relative risks since this is a cross-sectional study with exposure and prevalence of outcome measured at the same time point [16].

### Ethical considerations

This study is based on data routinely collected in the Nigerian public health sector, most of which are publically available. Some data were accessed from the National Health Management Information System [17]. Data not in the public domain were made available by the Federal Ministry of Health and the National PHC Development Agency upon request. Formal permission from the NPHCDA for the use of data not in public domain for the purpose of this research was obtained in writing. Approval to conduct this study was obtained from the Research Ethics Committee of the London School of Hygiene & Tropical Medicine. As all data used in the analysis are aggregated at the sub-national level and no individual or personal information was collected, local ethics approval was not required.

## Results

Data were available from 1,954 health facilities (343 and 464 in the Northern states of Jigawa and Kano; 525 and 622 of in the Southern states Ondo and Abia, respectively) in 106 local government areas. While all 18 local government areas in Ondo had available data from PHC reviews, data was available from 94% of local government areas in Abia state, 61% in Kano and 56%in Jigawa. Table 3 compares access to routine immunization between states in the same region while also making comparisons between the North and South. Table 4 compares trans-regional access between states of similar routine immunization coverage categories (High and Low coverages).

**Table 3:** Supply-side Indicators for routine immunization across selected States (Trans-regional), n (%).

INDICATORS	NORTH (N = 807)		P-VALUE*	SOUTH (N = 1147)		P-VALUE*	TOTAL
	**KANO (N = 464)**	**JIGAWA (N = 343)**		**ABIA (N = 622)**	**ONDO (N = 525)**		**P-VALUE** **
Percentage of Health Facilities without stock out of Oral Polio Vaccines or Pentavalent vaccine in the reporting period	411 (89)	330(96)	<0.001	527(84.7)	512 (98)	<0.001	0.38
Percentage of Health Facilities with at least two trained vaccinators	335 (72)	226 (66)	0.0633	444 (71)	337 (64)	0.0109	0.52
**INDICATORS**	**KANO (N = 6,889,851)**	**JIGAWA (N = 2,968,723)**		**ABIA (N = 3,099,314)**	**ONDO (N = 4,284,205)**		**P-VALUE** **
Percentage of population living within 5 km radius from immunization service points (Health Facilities and outreach locations)	3,847,395 (56)	2,396,411 (81)	<0.001	1,422,987 (46)	2,719,700 (64)	<0.001	< 0.001

Low coverage states: Abia (Southern Nigeria) and Kano (Northern Nigeria)

* Fisher’s exact test for a difference in proportion between health facilities in Northern states and between health facilities in Southern states, respectively

** Fisher’s exact test for a difference in proportions between facilities in Northern and facilities in Southern states.

**Table 4:** Supply-side Indicators for routine immunization across selected states by coverages, n (%).

	**LOW COVERAGE**		**P-VALUE** *	**HIGH COVERAGE**		**P-VALUE** *
**INDICATORS**	**KANO (N = 464)**	**ABIA (N = 622)**		**JIGAWA (N = 343)**	**ONDO (N = 525)**	
Percentage of Health Facilities without stock out of Oral Polio Vaccines or Pentavalent vaccine in the reporting period	411 (89)	527(84.7)	0.07	330(96)	512 (98)	0.31
Percentage of Health Facilities with at least two trained vaccinators	335 (72)	444 (71)	0.79	226 (66)	337 (64)	0.66
**INDICATORS**	**KANO (N = 6,889,851)**	**ABIA (N = 3,099,314)**		**JIGAWA (N = 2,968,723)**	**ONDO (N = 4,284,205)**	
Percentage of population living within 5 km radius from immunization service points (Health Facilities and outreach locations)	3,847,395 (56)	1,422,987 (46)	<0.001	2,396,411 (81)	2,719,700 (64)	<0.001

* Fisher’s exact test for a difference in proportion between facilities in Low coverage and between facilities in High coverage states, respectively.

### Trans-Regional Differences

Table 3 shows how the high coverage states of Jigawa and Ondo ranked higher than the lower coverage states with respect to vaccine availability. However in analysing disparities between Northern and Southern states, there was no significant association between geographical location and experiences of vaccine stock-outs. Further, there was no evidence of a significant association between geographical location and availability of trained vaccinators. Overall, there was evidence of a significant association between residing in Northern versus Southern Nigeria and accessing routine immunisation services within a 5 km radius: people residing in Northern Nigeria were 1.13 times more likely to live within 5 km of routine immunisation service delivery points than those residing in Southern Nigeria.

### Interstate Differences

Table 3 compares access to routine immunization within the regions and shows evident disparities across all indicators between individual states irrespective of regional affiliations. The exception is in the analysis of the relationship between the two Northern states with respect to availability of two trained vaccinators. Here there is weak evidence against the null hypothesis, suggesting no disparities between the relatively high and low coverage states in the North with respect to availability of trained vaccinators.

Table 4 compares access among states with similar coverage. The results provide evidence against the null hypothesis, suggesting no disparities between states with similar coverage in terms of vaccine stock-outs and availability of trained vaccinators. Furthermore, the two low coverage states—Kano and Abia –had higher proportions of facilities with adequate number of trained vaccinators than the higher coverage states of Ondo and Jigawa.

## Discussion

This study suggests that the availability of human resources and commodities at routine immunisation service delivery points are independent of geographical region (Northern or Southern Nigeria). However, residents in Northern Nigeria were more likely to have vaccination service delivery points located within 5km radius of their settlements compared to their counterparts in Southern Nigeria. In addition, the study shows a generally sub-optimal supply of services, particularly in terms of the availability of trained vaccinators and geographical coverage of routine immunisation services. Perhaps reflecting the autonomy of individual states within the federal structure of governance in Nigeria, disparities were evident between individual states irrespective of regional affiliation.

Nigeria operates a federal system of government in which the roles of the various tiers with respect to health is not clearly defined constitutionally. While Nigeria’s National Health Policy supports the devolution of PHC to the local governments, the federal constitution may be interpreted to imply that state governments are primarily responsible for PHC, leaving the extent of participation of Local Government Authorities at the discretion of individual state governments [18, 4, 19]. Such a complex system may have had implications on health service delivery, particularly routine immunization. This may explain why Nigeria, after climaxing with an average routine immunization coverage of 81.5% for all vaccines during the unitary military regime between 1988 and 1990, experienced a decline following devolution of power to states with the advent of democracy in 1999. Whereas comparable countries in the region with unitary systems of government, such as Ghana, Cote d’Ivoire and Senegal perform higher than the African continent’s average DTP coverage of 75% [20], countries operating federal system of government, including Nigeria, South Africa and Ethiopia, fall below average [21].

The inter-state disparity in vaccine availability may also be due to the vaccine supply chain in Nigeria, which operates a ‘push-pull’ system. This involves the federal government purchasing and distributing vaccines to the states, which are in turn responsible for “pushing” vaccines to local governments. At this lower level, health facilities are required to retrieve (“pull”) vaccines from their local government cold stores. Previous reports have shown that the weakest link in this supply chain is between local governments and service delivery points, with health facilities not having vaccine collection plans and consistently experiencing stock-outs of at least one antigen despite adequate supplies at the national and state levels [22]. In 2014 national reports, one state in the North and five in the South seemed to persistently have less than four weeks supply of vaccines [23]. This suggests disparity in favour of the North, which contrasts with our findings in this study. This seeming disparity may result from attempts at ensuring that the areas with the greater need get priority supply (vertical equity). Although the ongoing polio eradication efforts may have had significant impact on vaccination coverage, particularly in the North which houses all the high-risk states [24], our findings suggest that this did not result in differential stock-out rates across the regions. In town hall meetings conducted in 2012 across Nigeria, participants in both North and South commonly identified numerous constraints to accessing immunization services, including vaccine stock-outs at local government level and lack of funds for health facilities to facilitate the “pull” process from local government headquarters [25].

In line with current estimates of the distribution of mid-level health workers—nurses and community health extension workers—in Nigeria, our study shows no regional disparities in human resources trained on delivering routine immunisation services. Northern Nigeria has a density of mid-level health workers of 22.7 per 100,000 of population compared to 21.5 in Southern Nigeria [26]. However, 55% of training institutions for community health extension workers are located in the North, while 75% of schools of nursing are located in the South. Thus, the majority of health workers trained on guidelines for immunisation are trained in the South and deployed to fill gaps in the North [26]. This trans-regional migration of health workers may be partly responsible for the uniformity in the distribution of immunisation workforce. However, 30% of health facilities lack an adequate number of trained personnel for vaccination services, indicating that in some facilities non-qualified personnel may be involved in administering vaccines. Previous studies have demonstrated significant associations between nurse density and vaccination coverage, as opposed to availability of doctors for which there was no significant association [27]. In Nigeria, as in many other developing countries, immunization services at PHC centres are delivered mostly by mid-level health workers. This cadre is closest to the public and is the backbone of PHC. While the importance of health workers in service delivery is not disputed, the variation in association between the cadre of health workers and immunisation outcome is not yet widely acknowledged.

Immunisation policy in Nigeria aims to provide services within a 5km or 1 hour walking range. But physical access was found to be sub-optimal across selected states irrespective of geographical region. A previous study in Jigawa found the location of health facilities to be biased in favour of local government headquarters, which tend to be in urban or semi-urban communities [28]. Several studies have shown a direct relationship between geographical accessibility and utilization of health services such as a progressive decline in utilization with distance to health service delivery points, levelling off at 4km from the facilities [29]; and a 50% decline in utilization at distance range of 2-4km [29, 30, 31, 32]. This has important implications for equity, as people who live in the most remote areas also tend to belong to the most disadvantaged social groups [33, 34].

The challenges with removing barriers to access to routine immunisation in Nigeria are more profound at the local government level. Even though local governments have the weakest financial and technical capacity to implement primary health care, the design of the decentralised system of governance is such that they carry the greatest burden of service delivery. Nigeria’s current revenue allocation formula disburses 52.68% of national revenue to the federal government, state governments receive 26.72%, while 20.60% is shared among the 774 local governments [35]. Although this does not reflect health allocations directly, it is evident that the tier of government that provides basic services of primary health care and primary education receives the least allocation of resources. Furthermore, state and local government resources are pooled in a common account under the control of the state governments, which are constitutionally empowered to disburse funds as they deem fit. Consequently, local governments often are the most fund-deprived administrative tier. This system of governance and resource allocation is thus potentially responsible for the difference in supply of services across various states rather than across regions, as observed in this study.

In Nigeria, vaccines and injection supplies account for 25% of total costs of immunization, while other costs include salaries (47%), training (4%), transport (6%) and infrastructure (4%) [36]. The federal government is responsible for the vaccination costs, while states and local governments are in charge of salaries, training and capital costs, accounting for about 55–60% of total immunization expenditure. Transport costs are the shared responsibility of all tiers of government via the ‘push-pull system’, with the local governments bearing the greater burden as they finance the transportation of commodities to health facilities. Based on our findings, we recommend that, to improve access to routine immunization in Nigeria, a strategy hinged on strengthening the capacity and accountability of local governments will have to be evolved.

Limitations of this study include the use of secondary data from the routine data system, which is known to have poor data quality due to inadequate training of data officers, constraints with availability of data tools as well as data transmission platforms, resulting in fluctuations in data reporting rates. In spite of these challenges, routine data are the best available source on the implementation of the expanded programme on immunization and are used in health programming and policy making both centrally and at the lower levels of the health system. In addition, the results of the study are prone to selection bias as a purposive sampling technique was used to select the states. This implies that our results may not be representative of and generalizable to the entire country, particularly as each state operates its own unique health system at the local government level. However, we endeavoured to be transparent on the criteria on which study states were selected, and these may guide the application of our results beyond the sample states. Although data from all LGAs that conducted PHC reviews within each sampled state were analysed, the proportion of LGAs that conducted PHC reviews in each state varied, thereby presenting a potential selection bias. However, given that we studied the supply-side determinants only (which are largely centrally coordinated in each state), it is unlikely that the absence of data from non-participating LGAs will have considerable impact on the results. A further limitation is that the PHC review does not provide data disaggregated along socio-cultural lines, which would have been desirable to conduct multivariate analysis to adjust for this potential confounder.

## Conclusion

Nigeria operates a complex system of government, with significant implications for PHC service delivery, including routine immunisation services. Our findings however suggest that regional supply-side disparities may not explain disparities in routine immunisation coverage across Nigeria. This reinforces the earlier reported socio-cultural explanations for the differences in uptake of routine immunization between Northern and Southern Nigeria. However, disparities in supply-side indicators seem to exist across individual states irrespective of their geographical region. This is perhaps because in Nigeria, each state government is free to determine its own health care delivery system and allocate financial resources as it deems fit, without input from the federal government. The weakest links in Nigeria’s immunization system are local governments, which are responsible for the delivery of PHC services. This weakness is largely due to poor financing. Efforts to improve the supply of routine immunisation services require financial and technical support for local governments in ensuring that the supply chain for vaccine commodities functions optimally, that there are skilled human resources to deliver routine immunisation services, and that health facilities are equitably distributed within local governments. Using a survey-based design rather than routine data in future research may provide further evidence for comparisons.

## References

[1] Stokes-Prindle C., Wonodi C., Aina M., Oni G., Olukowi T., Pate M. A. et al, "Landscape Analysis of Routine Immunizaion in Nigeria: Identifying Barriers and Prioritizing Interventions," International Vaccine Access Centre (IVAC), Johns Hopkins University School of Public Health, Bloomberg, 2012.

[2] National Population Commission (NPC) [Nigeria] and ICF International, "Nigeria Demographic and Health Survey 2013," NPC & ICF International, Abuja, Nigeria, and Rockville, Maryland, USA, 2014.

[3] Antai D., "Inequitable childhood immunization uptake in Nigeria: a multilevel analysis of individual and contextual determinants," *BMC Infect Dis*, vol. 20, pp. 181–190, 2009. 10.1186/1471-2334-9-181 PMC2787508 19930573

[4] Federal Government of Nigeria (FGN), "Constitution of the Federal Republic of Nigeria," FGN, Abuja, 1999.

[5] Abimbola S., Negin J., Jan S. and Martiniuk A., "Towards People-Centred Health Systems: A Multi-Level Framework for Analyzing Primary Health Care Governance in Low—and Middle-Income Countires," *Health Policy and Planning*, vol. 29, pp. ii29–1139, 2014. 25274638 10.1093/heapol/czu069 PMC4202919

[6] Federal Government of Nigeria, "National Immunization Policy," National Primary Health Care Development Agency, Abuja, 2009.

[7] National Primary Health Care Developemnt Agency (NPHCDA), "Guidelines for the Training of Resource Persons and Implementation of Quarterly Primary Health Care Planning & Implementation Review (Bottleneck Analysis Methodology)," NPHCDA, Abuja, 2012.

[8] Okehie-Offoha M. U. and Sadiku M. N., Eds., Ethnic and Cultural Diversity in Nigeria, Africa World Press, 1996.

[9] Osaghae E. and Suberu R., "A history of identities, violence and stability in Nigeria.," Centre for Research on Inequality, Human Security and Ethnicity, University of Oxford, Oxford, 2005.

[10] M. Abdul, M. Onose, M. Tauhida, I. Monica, M. Voke-Ighorodje, O. Adeyeye et al, "An Analysis of the Socio-Economic Religious, Socio-Cultural, Environmental, Technological, Language and Educational Factors on Women’s Rights in Nigeria: The Case of Northern Nigeria," 2012.

[11] Babalola S. and Fatusi A., "Determinants of Use of maternal Health Services in Nigeria-Looking Beyond Individual and Household Factors," BMC, Pregnancy and Childbirth, vol. 9, no. 43, 2009. 10.1186/1471-2393-9-43 PMC2754433 19754941

[12] National Primary Health Care Development Agency (NPHCDA), "Nigeria Immunization Coverage Survey (NICS) 2010.," NPHCDA, Abuja, 2010.

[13] National Primary Health Care Development Agency, "Institutionalization of the Primary Health Care Reviews in Nigeria: Status and Progress," NPHCDA, Abuja, 2013.

[14] EIU Canback, "Canback Global Income Distribution Database (C-GIDD)," Available from https://www.cgidd.com/, Accessed 8th October, 2015.

[15] Federal ministry of Health (FMOH), "National Strategic Health Developement Plan 2010–2015," FMOH, Abuja, 2010.

[16] Caneiro I. and Howard N., Eds., Measures of Association and Impact. Introduction to Epidemiology, 2nd edn ed., Open University Press, 2011, pp. 28–29.

[17] Federal Ministry of Health, "National Health Management Information System".

[18] Federal Ministry of Health (FMOH), "Revised National Health Policy," Federal Ministry of Health, Abuja, Nigeria, 2004.

[19] M. D. Gupta, V. Gauri and S. Khemani, "Decentralized Delivery of Primary Health Services in Nigeria: Survey Evidence from the States of Lagos and Kogi," The World Bank, 2003.

[20] Centres for Disease Control and Prevention (CDC), "Global Routine Vaccination Coverage, 2013," CDC, Atlanta, 2013.

[21] World Health Organization (WHO), "WHO/UNICEF estimates of national immunization coverage," WHO, Geneva, 2014.

[22] World Health Organization, "Routine Immunization Programme of Nigeria Vaccine Audit Report 2012," WHO, Geneva, 2012.

[23] National Primary Health Care Development Agency, "Annual Report of the National Primary Healthcare Development Agency," NPHCDA, Abuja, 2013.

[24] Mangal T. D., Aylward R. B., Mwanza M., Gasasira A., Abanida E., Pate M. et al, "Key issues in the persistence of poliomyelitis in Nigeria: a case-control study," *Lancet Global Health*, vol. 2, no. 2, pp. 90–97, 2014. 10.1016/S2214-109X(13)70168-2 25104665

[25] International Vaccine Access Center [IVAC], "Nigeria 1st National Vaccine Summit: Town Hall Meeting Reports," IVAC, Bloomberg, 2012.

[26] Africa Health Workforce Observatory (AHWO), "Human Resources for Health Country Profile—Nigeria," WHO, Geneva, 2008.

[27] Anand S. and Bärnighausen T., "Health workers and vaccination coverage in developing countries: an econometric analysis," *The Lancet*, vol. 369, no. 9569, pp. 1277–1285, 14 4 2007. 10.1016/S0140-6736(07)60599-6 17434403

[28] Jaro I. and Ibrahim A., "The accessibility problems of primary health care to rural people in Jigawa State, Nigeria," Global Advanced Research Journal of Social Science (GARJSS), vol. 1, no. 4, pp. 72–76, 2012.

[29] Feikin D. R., Nguyen L. M., Adazu K., Ombok M., Audi A., Slutsker L. et al, "The impact of distance of residence from a peripheral health facility on pediatric health utilisation in rural western Kenya," Tropical Medicine & International Health, vol. 14, no. 1, p. 54–61, 2009. 19021892 10.1111/j.1365-3156.2008.02193.x

[30] Stock R., "Distance and the utilization of health facilities in rural Nigeria," *Social Science and Medicine*, vol. 17, p. 563–570., 1983. 6879255 10.1016/0277-9536(83)90298-8

[31] Muller I., Smith T., Mellor S., Rare L. and Genton B., "The effect of distance from home on attendance at a small rural health centre in Papua New Guinea," *International Journal of Epidemiology*, vol. 27, pp. 878–884, 1998. 9839747 10.1093/ije/27.5.878

[32] Baker J. B., Bazemore A. W. and Jacobson C. J., "Rapid assessment of access to primary care in remote parts of the developing world," *Field Methods*, vol. 20, no. 3, pp. 296–309, 2008.

[33] Hart J. T., "The Inverse Care Law," *The Lancet*, vol. 297, no. 7696, p. 405–412, 1971. 10.1016/s0140-6736(71)92410-x 4100731

[34] Pullan R., Freeman M., Gething P. and Brooker S., "Geographical Inequalities in Use of Improved Drinking Water Supply and Sanitation across Sub-Saharan Africa: Mapping and Spatial Analysis of Cross-sectional Survey Data," *PLoS Medicine*, vol. 11, no. 4, 2014. 10.1371/journal.pmed.1001626 PMC3979660 24714528

[35] Lukpata V., "Revenue allocation formulae in Nigeria: A continuous search," *International Journal of Public Administration and Management Research*, vol. 2, no. 1, pp. 32–38, 2013.

[36] Ojo K., Yisa I., Soyibo A., Olubajo L. and Schoen P., "Cost of Routine Immunization in Nigeria," *CHECOD Working Paper Series*, 2011.

